# Hospital variation in revision rates after primary knee arthroplasty was not explained by patient selection: baseline data from 1452 patients in the Danish prospective multicenter cohort study, SPARK

**DOI:** 10.1007/s00167-023-07374-3

**Published:** 2023-04-21

**Authors:** Anne Mørup-Petersen, Michael Rindom Krogsgaard, Mogens Laursen, Frank Madsen, Kristian Breds Geoffroy Mongelard, Lone Rømer, Matilde Winther-Jensen, Anders Odgaard

**Affiliations:** 1 Department of Orthopaedic Surgery, Copenhagen University Hospital Herlev and Gentofte, Gentofte Hospitalsvej 1, 2900 Hellerup, Denmark; 2 Department of Orthopaedic Surgery, Section for Sports Traumatology, Bispebjerg and Frederiksberg Hospital, Copenhagen, University of Copenhagen, Bispebjerg Bakke 23, 2400 Copenhagen, NV Denmark; 3 Department of Orthopaedic Surgery, Aalborg University Hospital, Hobrovej 18-22, 9000 Aalborg, Denmark; 4 Department of Orthopaedic Surgery, Aarhus University Hospital, Palle Juul-Jensens Boulevard 99, 8200 Aarhus N, Denmark; 5 Department of Radiology, Copenhagen University Hospital Herlev and Gentofte, Gentofte Hospitalsvej 1, 2900 Hellerup, Denmark; 6 Department of Radiology, Aarhus University Hospital, Palle Juul-Jensens Boulevard 99, 8200 Aarhus N, Denmark; 7 Center for Clinical Research and Prevention, Department of Data, Biostatistics and Pharmacoepidemiology, Bispebjerg and Frederiksberg Hospital, Copenhagen, University of Copenhagen, Nordre Fasanvej 57, 2000 Frederiksberg, Denmark; 8 Department of Orthopaedic Surgery, Rigshospitalet, Copenhagen University Hospital, Blegdamsvej 9, 2100 København Ø, Denmark; 9 Department of Clinical Medicine, University of Copenhagen, Copenhagen, Denmark

**Keywords:** Knee arthroplasty, Knee replacement, Epidemiology, Patient-reported outcome measures, Revision rate variation, Regional difference, Hospital variation, Radiographic classification, Patient selection, Osteoarthritis

## Abstract

**Purpose:**

Revision rates following primary knee arthroplasty vary by country, region and hospital. The SPARK study was initiated to compare primary surgery across three Danish regions with consistently different revision rates. The present study investigated whether the variations were associated with differences in the primary patient selection.

**Methods:**

A prospective observational cohort study included patients scheduled Sep 2016 Dec 2017 for primary knee arthroplasty (total, medial/lateral unicompartmental or patellofemoral) at three high-volume hospitals, representing regions with 2-year cumulative revision rates of 1, 2 and 5%, respectively. Hospitals were compared with respects to patient demographics, preoperative patient-reported outcome measures, motivations for surgery, implant selection, radiological osteoarthritis and the regional incidence of primary surgery. Statistical tests (parametric and non-parametric) comprised all three hospitals.

**Results:**

Baseline data was provided by 1452 patients (89% of included patients, 56% of available patients). Patients in Copenhagen (Herlev-Gentofte Hospital, high-revision) were older (68.6 ± 9 years) than those in low-revision hospitals (Aarhus 66.6 ± 10 y. and Aalborg (Farsø) 67.3 ± 9 y., *p* = 0.002). In Aalborg, patients who had higher Body Mass Index (mean 30.2 kg/m^2^ versus 28.2 (Aarhus) and 28.7 kg/m^2^ (Copenhagen), *p* < 0.001), were more likely to be male (56% versus 45 and 43%, respectively, *p* = 0.002), and exhibited fewer anxiety and depression symptoms (EQ-5D-5L) (24% versus 34 and 38%, *p* = 0.01). The preoperative Oxford Knee Score (23.3 ± 7), UCLA Activity Scale (4.7 ± 2), range of motion (Copenhagen Knee ROM Scale) and patient motivations were comparable across hospitals but varied with implant type. Radiological classification ≥ 2 was observed in 94% (Kellgren-Lawrence) and 67% (Ahlbäck) and was more frequent in Aarhus (low-revision) (*p* ≤ 0.02), where unicompartmental implants were utilized most (49% versus 14 (Aalborg) and 23% (Copenhagen), *p* < 0.001). In the Capital Region (Copenhagen), the incidence of surgery was 15–28% higher (*p* < 0.001).

**Conclusion:**

Patient-reported outcome measures prior to primary knee arthroplasty were comparable across hospitals with differing revision rates. While radiographic classifications and surgical incidence indicated higher thresholds for primary surgery in one low-revision hospital, most variations in patient and implant selection were contrary to well-known revision risk factors, suggesting that patient selection differences alone were unlikely to be responsible for the observed variation in revision rates across Danish hospitals.

**Level of evidence:**

II, Prospective cohort study.

## Introduction

**Fig. 1:**
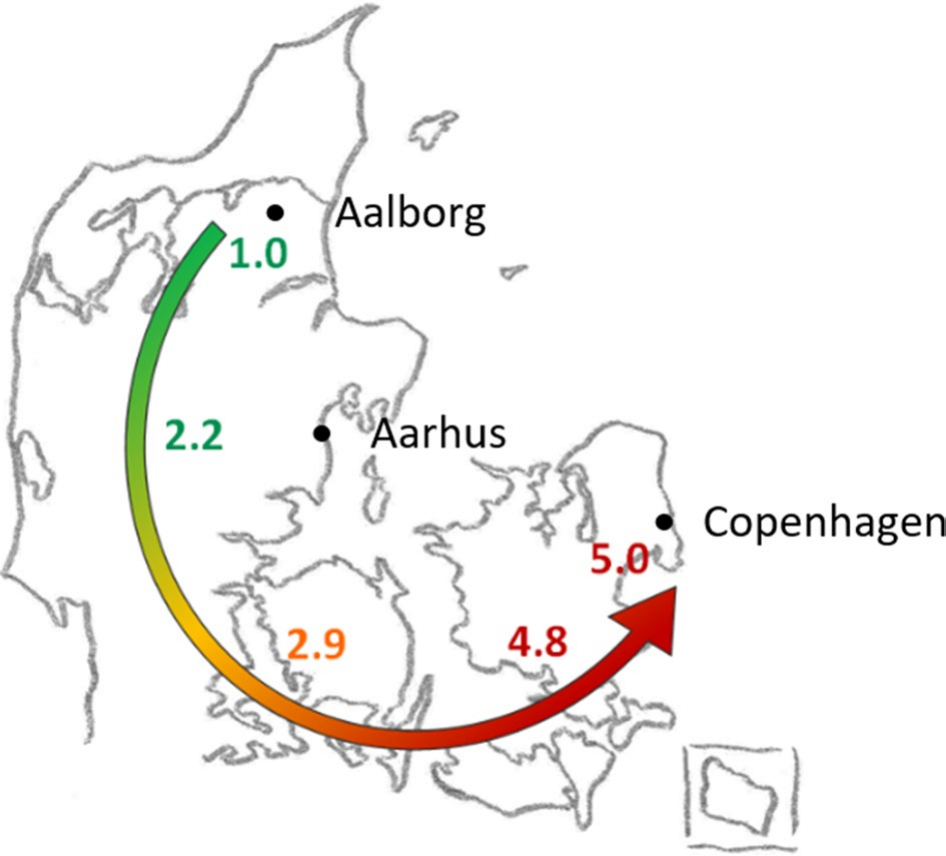
2-year revision rates, Danish regions. Overview of 2-year revision rates after primary knee arthroplasty in the Danish regions (The Danish Knee Arthroplasty Register, Annual Report 2016). The three study hospitals are mapped

Assessment of the quality of knee arthroplasty (KA) surgery is traditionally based on cumulative revision rates (CRR) [29]. According to data from national arthroplasty registries, there are significant CRR differences between countries and large and statistically significant differences within countries and between hospitals [7, 33]. These observations are rarely discussed and attempts to explain the variation often focus on implant selection. Data from the Danish Knee Arthroplasty Register show a statistically significant variation across the five administrative regions in Denmark for 1-, 2-, 5- and 10-year cumulative revision rates (CRRs) [42]. The CRR of the Capital Region has persistently been the largest and lower rates have been seen with increasing distance from the capital, Copenhagen (Fig. 1). For instance, in 2015 when this study was initiated, the 2-year CRR was 5.0% in the Capital Region, 2.2% in Central Denmark Region and 1.0% in North Denmark Region [42]. Variations among regions or hospitals can occur by chance, but consistent differences in CRRs could indicate systematic differences in the indications for the primary procedure, patient demographics, the quality of surgery including implant selection, or indications for revisions – or combinations of these. Demographics, preoperative knee symptoms and the severity of radiographic knee osteoarthritis (OA) are all factors that are associated with the degree of postoperative patient satisfaction and the risk of revision [6, 11, 14, 15, 29, 35]. These variables, however, have not specifically been compared across hospitals with varying revision rates following KA.

For the three Danish regions in issue, register data provided no explanation for the CRR differences, and apart from undocumented assertions of cultural differences between regions, there were no hypotheses regarding the factors that might be responsible. This motivated the initiation of the prospective observational cohort study, SPARK (“Variation in patient Satisfaction, Patient-reported outcome measures, radiographic signs of Arthritis, and Revision rates in Knee arthroplasty patients in three Danish regions”). The present part of the SPARK study aims to compare patient characteristics, knee radiographs, implant selection and patient-reported outcome measures (PROMs) obtained before primary KA in a large hospital of each region, and to investigate whether hospital variations in patient selection were associated with the CRR differences. Postoperative outcomes will be reported in a separate publication.

## Materials and methods

The National Committee of Health Research Ethics provided ethical approval (Protocol no. 16038343, 2 September 2016) and all patients gave their written consent to participate. Reporting adheres to the STROBE guidelines for observational cohort studies.

### Patient inclusion

This prospective observational cohort study invited the largest knee arthroplasty university hospital in each of the three Danish regions that differed most in revision rates after KA surgery: Aarhus University Hospital in the Central Denmark Region, Aalborg University Hospital Farsø in the North Denmark Region and Copenhagen University Hospital Herlev-Gentofte in the Capital Region. Revision rates for each of the three hospitals were comparable to those for the region as a whole (Table 1) [42]. All hospitals were public (94% of primary KA’s were performed in public hospitals in 2017) [43].

**Table 1:** Latest cumulative revision rates in study hospitals and according regions at study start in 2016 (means of preceding 3 years)

Hospital (region)	2-year CRR (%) (Primary surgery 2011–13)	5-year CRR (%) (Primary surgery 2008–10)
Aarhus University Hospital (Central Denmark Region)	1.9 (2.5)	4.3 (4.1)
Aalborg University Hospital Farsø (North Denmark Region)	1.6 (1.5)	5.3 (4.6)
Copenhagen University Hospital Herlev-Gentofte (The Capital Region)	**5.6 (4.7)**	**12.0 (7.5)**

Data from the Danish Knee Arthroplasty Register, Annual Report 2016. Bold figures denote the highest cumulative revision rate (CRR) of each year

From 1 September 2016 to 31 December 2017, patients who were scheduled for primary KA, i.e., total (TKA), medial/lateral unicompartmental (MUKA/LUKA) or patellofemoral arthroplasty (PFA) were eligible for inclusion. Participation did not interfere with implant selection or surgical routines. Exclusion criteria were knee tumors, hemophilia, severe developmental lower limb deformities, dementia or language barriers that could not be overcome by help from relatives. Patients unable to answer questionnaires online were excluded, with the exception of the final 6 months of the inclusion period (July 2017-Dec 2017) during which participation via paper questionnaires was permitted.

Patients were recruited for the study by the surgeon (Aarhus and Aalborg) or by an employed medical student (Copenhagen). Two days later, patients received an email with a unique link to the preoperative PROM set or a letter with the same content. Up to two email reminders were sent, three days apart, if necessary. To avoid confusion among patients with bilateral knee trouble, the email specified that the knee scheduled for surgery was the object of the study. Patients planned for surgery on both knees could participate twice if the operations were conducted on separate occasions, while patients with simultaneous bilateral surgery were asked to choose which knee to participate with in advance [24]. Since PROMs were the cornerstone of this study, patients who failed to complete the questionnaire prior to surgery were excluded.

Post-hoc quantification of inclusion rates and demographic comparisons between participants and non-participants were conducted. As the time from inclusion to surgery varied, these analyses were based upon registered surgical activity during a certain time period (1 Jan to 31 Dec 2017) [36].

### Patient-reported outcome measures (PROMs) at baseline

Knee-specific PROMs included Oxford Knee Score (OKS, 0–48, 48 best) as the primary outcome [3, 5, 24, 40], UCLA Activity Scale (0–10, 10 highest) [23, 37] and Copenhagen Knee ROM Scale (CKRS) assessing patient-reported passive range of knee motion (flexion 0–6 (6 max), extension 0–5 (5 max))^1^ [21, 22]. All knee-specific questions were preceded by the generic EQ-5D-5L and EQ-VAS [41, 44] and a global knee anchor question, “How is your knee?” (Visual Analogue Scale (VAS) from “My knee does not work at all or is extremely painful” to “My knee is pain-free and functions normally”, 0–100, 100 best). Patients’ motivation for the surgery was evaluated by marking up to 5 of 13 common reasons provided, based on explorative interviews with 35 knee OA patients (unpublished) or adding one free-text reason.

Patients reported their height and weight as well as additional health and lifestyle information, including their degree of urbanization (”city/suburb”, “small town/village” or “countryside”), daily smoking (“yes”/”no”), and alcohol consumption (more or less than two standard drinks (12 g alcohol) per day). Patients were asked whether the knee was their main physical disability, and “How often do you take painkillers due of your knee?” with five answer options ranging from “more than once per day” to “rarely or never” (full wording in Table 3).

### Radiographic classification of knee osteoarthritis

The severity of tibiofemoral OA was assessed in blinded preoperative weight-bearing postero-anterior knee radiographs with the knee flexed 15–30° [2]. Patients listed for PFA or LUKA and those with predominantly lateral OA on radiographs were excluded from this analysis because the radiographic basis for surgery could not be fairly assessed without tangential (Skyline, Merchant) or flexed (Rosenberg) views, respectively.

Two radiologists with expertise in musculoskeletal radiology viewed the radiographs in a random sequence. First, the Ahlbäck classification (0–5, 5 severe), and secondly, in a new round of random order, the Kellgren-Lawrence classification (K-L, 0–4, 4 severe) was recorded for each patient [1, 13, 17]. In case of disagreement, both radiologists reevaluated each radiograph together and reached a consensus. Using a novel heuristic-based method, radiographs were evaluated free of classifications by 13 experienced knee arthroplasty surgeons from all five Danish regions. Each surgeon was presented with the knee radiographs in pairs and was asked to choose the radiograph that they expected would cause the most severe knee symptoms, *not* considering any formal grading system but instead using their personal experience and heuristics, i.e., “rule-of-thumb”. These thousands of comparisons resulted in a complete ranking of all radiographs [28].

### Incidence of surgery and implant selection

The incidence of primary KA on a regional level was retrieved from the National Patient Register by NOMESCO procedure code KNGB (age > 40 years and subgroup 60–79 years). The CRRs for the hospitals (Table 1) were retrieved from the Danish Knee Arthroplasty Register (97% completeness). On an individual level, the medical record was consulted in case of a mismatch in laterality or implant type from inclusion to postoperative registration.

### Statistics

Sample size and inclusion period were determined by clinical relevance and feasibility. Throughout the study period, around 1800 operations were anticipated and with a 75% inclusion rate and 80% response rate, 1080 responses would be ready for analysis. Any regional variations that were not detectable in a sample of this size were considered clinically irrelevant to the overall study question.

All analyses were based on the null hypothesis that patient selection was identical across the three hospitals. Due to the explorative nature of the study, additional data-driven analyses were allowed [30]. All tests were unpaired as though each knee belonged to a unique participant [32]. OKS and EQ-5D data were treated as numeric variables [24], as were knee flexion and extension [21], while Ahlbäck, K-L, surgeons’ ranking, and UCLA ratings were ordinal. A separate article describes the statistical details of heuristics-based assessment of radiographs using the Bradley-Terry model for paired comparisons [28].

Unless otherwise specified, statistical tests compared all three centres, *not* one against the mean. The significance of difference tests depended on the type and structure of data: Chi-square test for dichotomized variables, unpaired t-test or one-way analysis of variance (ANOVA) for parametric variables and Mann–Whitney *U* or Kruskal–Wallis test (> 2 groups) for nonparametric (ordinal) data. General linear regression models were used to estimate the effects of independent numerical variables on dependent variables, and when adjustment for confounders was relevant, multiple linear regression analyses were conducted (noted in text). Aarhus was selected as the reference hospital as it was situated between the two other hospitals in terms of geography, urbanization and CRR, i.e., the disparities known prior to inclusion. The level of significance was set to 0.05 (two-sided) and 95% confidence intervals (CI) were supplied when relevant. Data collection and Case Report Forms etc. were handled by Procordo Software Aps, Copenhagen. In Mar 2019, analyses were conducted using R (RStudio) [31].

## Results

### Patient inclusion

**Fig. 2:**
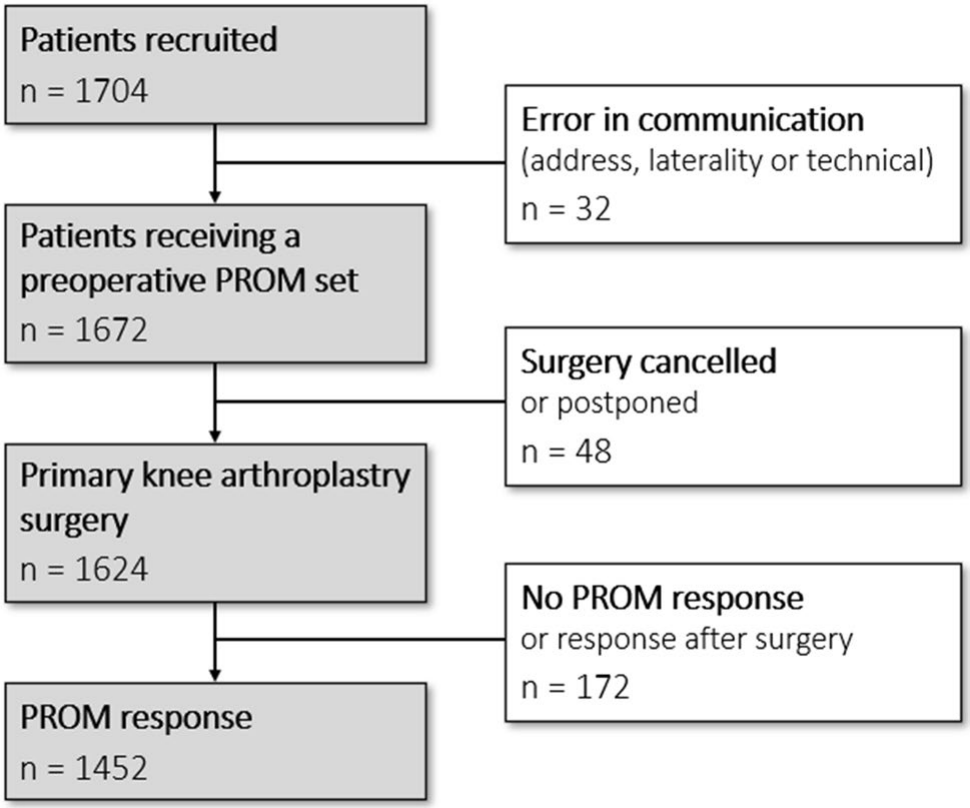
Inclusion flowchart. See text for details

Questionnaires were sent to 1704 patients (Fig. 2), 52 of those through letter. In 32 cases, the email address or laterality was wrong, or a technical error occurred, and 48 patients had their procedure cancelled or postponed beyond the research period. Consequently, 1624 patients received a questionnaire, and 1452 patients (89%) completed the PROM set at a mean of 29 days before surgery, spending an average of 12:30 min each patient. The 53 patients who participated with separate knees accounted for 7.3% of responses.

In the post-hoc inclusion analysis, 1924 patients were operated in 2017 at the three hospitals, 1083 of whom (56%) provided PROM data for this study; 62% in Aarhus, 38% in Aalborg and 62% in Copenhagen (Table 2). Non-responding participants were evenly distributed across hospitals (Aarhus 7.0%, Aalborg 8.2% and Copenhagen 10.0%, *p* = 0.2, Chi-square). In the total 2017 patient population, Copenhagen patients were older (mean 68.8 y.) than those at the other two hospitals (Aarhus 67.1 and Aalborg 67.6 y., *p* = 0.006, ANOVA). In Aalborg, there were more male patients (48%) than in Aarhus (39%) and Copenhagen (38%), (*p* = 0.005, Chi-square). The proportion of male inhabitants aged 60–79 years in each region ranged from 47% (Central Denmark and Capital Region) to 49% (North Denmark Region) [36].

**Table 2:** Inclusion analysis based on complete surgical activity in 2017

	Complete primary KA population 2017	SPARK participation		
		Yes	No	*P*
Patients (*n* (%))				
Total	1924 (100)	1083 (56)	841 (44)	–
Aarhus	391 (100)	243 (62)	148 (38)	–
Aalborg	429 (100)	161 (38)	268 (63)	–
Copenhagen	1104 (100)	679 (62)	425 (38)	–
Age (mean ± SD)				
Total	68.2 ± 9.8	67.7 ± 9.2	68.8 ± 10.5	**0.020**^*1*^
Aarhus	67.1 ± 10.6	66.1 ± 9.9	68.7 ± 11.5	**0.019**^*1*^
Aalborg	67.6 ± 9.8	66.7 ± 8.8	68.2 ± 10.3	0.141^a^
Copenhagen	68.8 ± 9.4	68.5 ± 9.0	69.2 ± 10.2	0.256^a^
Male sex (*n* (%))				
Total	779 (41)	459 (42)	320 (38)	**0.016**^*2*^
Aarhus	153 (39)	105 (43)	48 (32)	**0.043**^*2*^
Aalborg	202 (48)	83 (52)	119 (44)	0.070^b^
Copenhagen	424 (38)	271 (40)	153 (36)	0.125^b^

Bold figures denote significant values of *p* < 0.05

*P* values < 0.05 indicate a skewness in the distribution of study participants and non-participants based on surgical activity in 2017

*KA* Knee Arthroplasty. SPARK (study):” Variation in patient Satisfaction, Patient-reported outcome measures, radiographic signs of Arthritis, and Revision rates in Knee arthroplasty patients in three Danish regions”

^a^Unpaired *t* test

^b^Chi-Square test

In the SPARK cohort, males and younger patients more often agreed to participate in the SPARK study than females and older patients (Table 2). Further analyses (not shown) found that the distribution of implant types within each hospital did not differ between participants and non-participants (*p* ≥ 0.2, Chi-square).

SPARK participants from Copenhagen had a mean age of 68.6 years, 1.4–2.0 years older than those in Aalborg (67.3 y.) and Aarhus (66.6 y.), respectively (*p* = 0.002, ANOVA) (Table 3). Male sex was more prevalent in Aalborg (56%) than in Copenhagen (43%) and Aarhus (45%) (*p* = 0.002, Chi-square). In Aalborg, males (68.8 y.) were 3.5 years older than females (65.3 y.) (CI 1–6, *t* test), whereas in the other hospitals, there was no significant difference. BMI (mean 29.5 ± 5 kg/m^2^) was lower in the elderly (− 0.13 kg/m^2^/year, CI − 0.16-(− 0.11), linear regression) and higher in females (+ 0.69 kg/m^2^, CI 0.2–1.2) as well as in Aalborg patients (+ 1.5–1.7 kg/m^2^, *p* < 0.001), even after adjusting for age and sex (+ 1.4–1.9 kg/m^2^, adjusted). There were no differences between hospitals for smoking, alcohol consumption, physical activity level (UCLA) or self-reported general health (EQ Index and VAS). Except for smoking, males reported significantly higher levels of these parameters (Table 3) as compared to females (significant on hospital level for ULCA and alcohol consumption, only). In sub-analyses of EQ-5D-5L items, 76% of Aalborg patients were “neither anxious or depressed”, compared to 66% in Aarhus and 62% in Copenhagen (*p* = 0.01, Kruskal–Wallis). This hospital difference was only significant among females (*p* females = 0.03, males = 0.3). The 41 patients who responded by letter (75.9 y) were 8.1 years older than those who responded via email (67.8 y) (CI 6–10, t-test) and 29 (71%) were female (54% in the email group, p = 0.05, Chi-square).

**Table 3:** Preoperative data from 1452 responding patients

	Total sample	Hospital				Sex		*p*
		Aarhus (Low rev.rate)	Aalborg (Low rev.rate)	Copenhagen (High rev.rate)	*P*	Female	Male	
Patients (%)	1452 (100)	321 (22)	202 (14)	929 (64)		793 (55)	659 (45)	
**Demography & implants**								
Age [range]	68.0 ± 9.3 [29–93]	66.6 ± 9.7 [29–89]	67.3 ± 9.1 [41–89]	68.6 ± 9.1 [37–93]	**0.002**^5^	67.7 ± 9.7 [29–93]	68.3 ± 8.7 [43–89]	0.2^6^
Male sex (%)	659 (45)	145 (45)	114 (56)	400 (43)	**0.002**^7^	0 (0)	659 (100)	–
Implant type (%)					** < 0.001**^7^			**0.001**^7^
TKA	1059 (73)	164 (51)	174 (86)	721 (78)		590 (74)	469 (71)	
MUKA	336 (23)	129 (40)	25 (12)	182 (20)		160 (20)	176 (27)	
PFA	50 (3.4)	23 (7.2)	3 (1.5)	24 (2.6)		38 (4.8)	12 (1.8)	
LUKA	7 (0.5)	5 (1.6)	0 (0.0)	2 (0.2)		5 (0.6)	2 (0.3)	
Health & lifestyle*								
Weight (kg)	86 ± 17	85 ± 17	90 ± 15	85 ± 17	**0.002**^5^	81 ± 16	92 ± 15	** < 0.001**^6^
BMI (kg/m^2^)	29 ± 5.0	28.5 ± 4.6	30.2 ± 5.1	28.7 ± 5.1	** < 0.001**^5^	29.2 ± 5.7	28.5 ± 4.1	**0.009**^6^
BMI group (%)					** < 0.001**^8^			0.6^9^
Normal (< 25)	329 (23)	77 (24)	26 (13)	226 (24)		196 (25)	133 (20)	
Overweight (25–29.9)	589 (41)	140 (44)	78 (39)	371 (40)		284 (36)	305 (46)	
Obese (≥ 30)	529 (37)	102 (32)	98 (49)	329 (36)		309 (39)	220 (3)	
Alcohol (> 2 units per day) (%)	164 (11)	36 (11)	15 (7)	113 (12)	0.2^7^	43 (5)	121 (18)	** < 0.001**^7^
Daily smoking (%)	159 (11)	41 (13)	21 (10)	97 (11)	0.5^7^	87 (11)	72 (11)	1^7^
Degree of urbanization (%)					** < 0.001**^8^			0.8^9^
Countryside	78 (5)	18 (6)	33 (16)	27 (3)		43 (5)	35 (5)	
Small town or village	354 (24)	75 (23)	111 (55)	168 (18)		190 (24)	164 (25)	
City or suburb	1019 (70)	228 (71)	58 (29)	733 (79)		559 (71)	460 (70)	
Participation by letter (%)	41 (3)	5 (1.6)	10 (5.0)	26 (2.8)	0.1^7^	29 (3.7)	12 (1.8)	0.05^7^
EQ-VAS	61 ± 22	62 ± 21	58 ± 24	62 ± 22	0.1^5^	59 ± 22	65 ± 21	** < 0.001**^6^
EQ-5D-5L Index	0.59 ± 0.2	0.59 ± 0.15	0.61 ± 0.12	0.59 ± 0.15	0.1^5^	0.58 ± 0.15	0.60 ± 0.14	**0.03**^6^
UCLA Activity Scale	4.7 [4] ± 2	4.8 [4] ± 1.9	4.8 [4] ± 1.9	4.7 [4] ± 1.8	0.6^8^	4.5 [4] ± 1.7	5.1 [5] ± 2.0	** < 0.001**^9^
Knee-specific PROMs*								
OKS	23.3 [24] ± 7	23.5 [24] ± 7.0	23.2 [24] ± 6.5	23.3 [23] ± 6.7	0.9^5^	22.0 [22] ± 6.4	24.8 [25] ± 6.8	** < 0.001**^6^
Global knee anchor	28 ± 18	27 ± 17	30 ± 18	29 ± 18	0.2^8^	28 ± 18	29 ± 18	0.1^9^
Range of motion (Copenhagen Knee ROM Scale)^1^								
Flexion (CKRS)	4.9 [5] ± 1.2	4.8 [5] ± 1.2	4.8 [5] ± 1.1	4.9 [5] ± 1.2	0.2^5^	4.9 [5] ± 1.2	4.9 [5] ± 1.2	0.6^6^
Deficit (CKRS 0–4) (%)	416 (29)	97 (30)	58 (29)	261 (28)	0.7^7^	223 (28)	193 (29)	0.7^7^
Extension^2^ (CKRS)	3.5 [4] ± 1.0	3.4 [4] ± 1.0	3.4 [4] ± 0.9	3.5 [4] ± 0.9	0.2^5^	3.5 [4] ± 1.0	3.5 [4] ± 0.9	0.4^6^
Deficit (CKRS 0–3) (%)^2^	340 (49)	63 (45)	72 (62)	205 (46)	**0.007**^7^	200 (49)	140 (49)	1^7^
My knee is my main disability (%)	1261 (87)	289 (90)	176 (87)	796 (86)	0.1^7^	680 (86)	581 (88)	0.3^7^
Analgesics due to knee pain (%)					0.09^8^			** < 0.001**^9^
More than once perday	667 (46)	145 (45)	83 (41)	439 (47)		407 (51)	260 (40)	
Once per day	187 (13)	34 (11)	32 (16)	121 (13)		95 (12)	92 (14)	
More than once per week	218 (15)	42 (13)	27 (13)	149 (16)		129 (16)	89 (14)	
More than once per month	142 (10)	39 (12)	19 (9)	84 (9)		70 (9)	72 (11)	
Rarely or never	237 (16)	61 (19)	41 (20)	135 (15)		91 (11)	146 (22)	
Degree of radiographic OA^3^								
*n* (%)	1051 (100)	206 (20)	171 (16)	674 (64)		538 (51)	513 (49)	
K-L classification (%)					**0.02**^8^			**0.01**^9^
0	7 (1)	0 (0.0)	2 (1.2)	5 (0.7)		5 (0.9)	2 (0.4)	
1	57 (5)	4 (1.9)	13 (7.6)	40 (5.9)		35 (6.5)	22 (4.3)	
2	136 (13)	24 (12)	23 (14)	89 (13)		76 (14)	60 (12)	
3	787 (75)	160 (78)	123 (72)	504 (75)		395 (73)	392 (76)	
4	64 (6)	18 (8.7)	10 (5.8)	36 (5.3)		27 (5.0)	37 (7.2)	
K-L classification ≥ 2 (%)	987 (94)	202 (98)	156 (91)	629 (93)	**0.01**^7^	498 (93)	489 (95)	0.08^7^
K-L classification ≥ 3 (%)	851 (81)	178 (86)	133 (78)	540 (80)	0.07^7^	422 (78)	429 (84)	**0.04**^7^
Ahlbäck score (%)					0.1^8^			**0.01**^9^
0	56 (5)	7 (3)	9 (5)	40 (6)		37 (7)	19 (4)	
1	289 (28)	44 (21)	56 (33)	189 (28)		158 (29)	131 (26)	
2	401 (38)	94 (46)	61 (36)	246 (37)		198 (37)	203 (40)	
3	291 (28)	57 (28)	43 (25)	191 (28)		140 (26)	151 (29)	
4	12 (1)	3 (1.5)	2 (1.2)	7 (1.0)		4 (0.7)	8 (1.6)	
5	2 (0)	1 (0.5)	0 (0.0)	1 (0.1)		1 (0.2)	1 (0.2)	
Ahlbäck score ≥ 2 (%)	704 (67)	154 (75)	106 (62)	444 (66)	**0.02**^7^	342 (64)	362 (71)	**0.02**^7^
Ahlbäck score ≥ 3 (%)	305 (29)	61 (30)	45 (26)	199 (30)	0.7^7^	145 (27)	160 (31)	0.1^7^
Surgeons’ ranking^4^ (mean [25–75%])	540 [270–808]	380 [188–718]	598 [315–864]	561 [293–824]	** < 0.001**^8^	575 [318–845]	503 [238–778]	**0.002**^9^

Bold figures denote significant values of *p* < 0.05

^*^Patient-reported data. When nothing else is stated, means [and medians] ± SD are reported. For some non-parametric variables, means and ± SD have been reported to aid comparison

*TKA* Total Knee Arthroplasty. *MUKA/LUKA* Medial/Lateral Unicompartmental Knee Arthroplasty. *PFA* Patellofemoral Arthroplasty. *BMI* Body Mass Index (BMI group “underweight” (< 18.5 kg/m^2^) comprised only 2 patients, who were thus included in the “normal” group). *OKS* Oxford Knee Score (0–48 version, 48 best). UCLA Activity Scale range 1–10 (10 most active). *Global knee anchor* Patients’ overall knee assessment, recorded on VAS (0–100, 100 best). *OA* osteoarthritis. *K-L* Kellgren-Lawrence

^1^Copenhagen Knee ROM Scale: Flexion 0–6 (6 is max), Extension 0–5 (5 is max), see text for details. ^2^*n* = 699. ^3^*n* = 1051. ^4^Surgeons’ ranking: radiographic knee OA severity, total range 1- 1051 (1 is most severe)

Statistical tests: ^5^One-way analysis of variance (ANOVA). ^6^unpaired *t* test.  ^7^Chi-square test  ^8^Kruskal–Wallis test  ^9^Mann–Whitney *U* test

### Patient-reported outcome measures (PROMs) at baseline

**Fig. 3:**
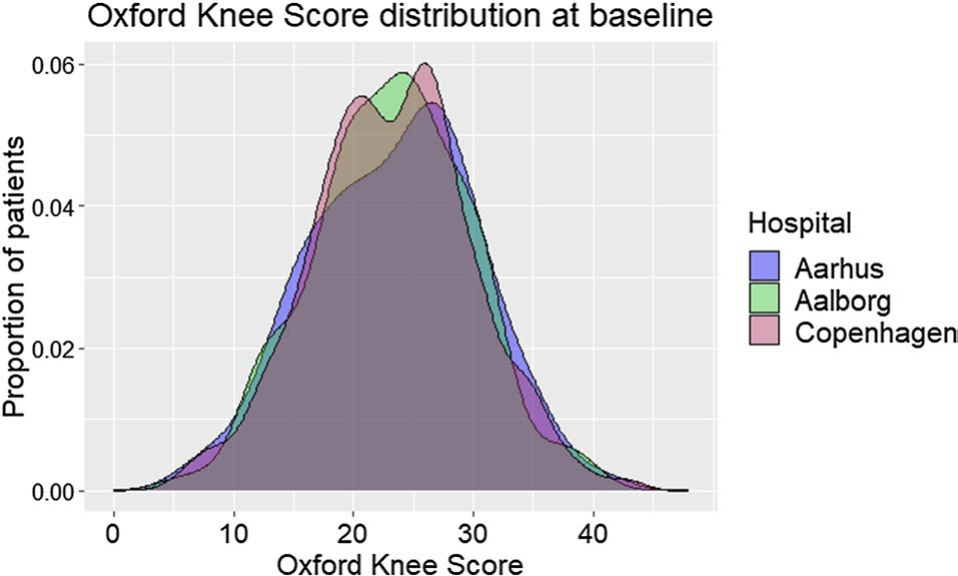
Oxford Knee Score at baseline. Distribution of preoperative Oxford Knee Score per hospital (Kernel density plot)

OKS at baseline did not differ among patients in the three hospitals (23.3 ± 7, *p* = 0.9, ANOVA) (Table 3, Fig. 3), even after adjusting for age, sex and BMI (multiple linear regression). The same was true for use of analgesics, knee flexion and the global knee anchor (Table 3). Extension deficits were more prevalent in Aalborg (62 vs. 45–46%, *p* = 0.007, Chi-square). Males scored 2.8 OKS points higher than females in all hospitals (CI 2–4, *t* test) and reported less frequent use of analgesics, while the sex difference in the overall perception of the knee condition (global knee anchor) was not significant (*p* = 0.1, Mann–Whitney *U*). OKS was significantly lower (− 2.6 points, CI − 3−(− 2), t-test) in obese patients (BMI > 30) and in smokers (− 1.5 points, CI − 3−(− 0.4), *t* test). There were no hospital differences in patients’ motivations for surgery (*p* ≥ 0.1, Chi-square), but stratification by implant and sex revealed significant variation (Table 4).

**Table 4:** Patients’ motivations for surgery (total SPARK cohort)

	Total	TKA	MUKA	*p*^1^	Females	Males	*p*^1^
*n* (%)	1452	1059 (73)	336 (23)		793 (55)	659 (45)	
Pain	1174 (82)	843 (81)	288 (88)	**0.009**	649 (84)	525 (81)	0.1
Mobility (walking, stairclimbing, bicycling)	784 (55)	592 (57)	159 (48)	**0.008**	450 (58)	334 (51)	**0.01**
Sports, exercise & physical activity	580 (41)	414 (40)	140 (43)	0.4	298 (39)	282 (43)	0.07
Knee motion and stability	521 (37)	392 (38)	107 (33)	0.1	290 (38)	231 (36)	0.5
The surgeons’ advice	516 (36)	376 (36)	117 (36)	0.9	276 (36)	240 (37)	0.6
Hobbies (leisure time, travelling)	474 (33)	347 (33)	113 (34)	0.8	236 (31)	238 (37)	**0.02**
Mood and energy	471 (33)	337 (32)	115 (35)	0.4	273 (35)	198 (31)	0.06
Tired of taking medication	404 (28)	281 (27)	106 (32)	0.08	246 (32)	158 (24)	**0.002**
Duties (housework, gardening, helping others)	440 (31)	330 (32)	98 (30)	0.6	221 (29)	219 (34)	**0.04**
Independency and selfcare	390 (27)	310 (30)	72 (22)	**0.006**	239 (31)	151 (23)	**0.002**
Work	242 (17)	156 (15)	75 (23)	**0.001**	117 (15)	125 (19)	**0.047**
Being with family and friends	176 (12)	141 (14)	32 (10)	0.08	111 (14)	65 (10)	**0.02**
Marital (incl. sexual) life	66 (5)	46 (4)	17 (5)	0.7	27 (4)	39 (6)	**0.03**
Missing answer	16 (1)	11 (1)	4 (1)	–	13 (2)	3 (1)	**–**

Bold figures denote significant values of *p* < 0.05

Answers to the question,”Which factors or problems made you choose surgery? Pick up to 5 motivations”. Options are listed by overall frequency. *TKA* Total Knee Arthroplasty. *MUKA* Medial Unicompartmental Knee Arthroplasty. ^1^Chi-Square test

### Radiographic classification of knee osteoarthritis

Exclusions were made for 50 PFA, 7 LUKA patients and 167 patients with predominantly lateral OA. 177 radiographs were unavailable due to logistical matters unrelated to the patient, leaving 1051 radiographs (86% of those possible) ready for analysis. The two radiologists reached a moderate interobserver agreement of 0.59 (weighted Kappa) for both K-L and Ahlbäck [17]. Prior to consensus, they disagreed in 29% (K-L) and 41% (Ahlbäck) of cases, respectively. The surgeons’ heuristics-based evaluations (17,767 comparisons) ranked all radiographs from number 1 (most severe) to number 1051 [28].

Knee OA severity was unevenly distributed across hospitals according to K-L classification and surgeons’ ranking (Table 3). Mild degrees of knee OA (K-L/Ahlbäck < 2) were less prevalent in Aarhus patients (*p* = 0.01 (K-L), *p* = 0.01 (Ahlbäck), Chi-square test), and surgeons’ ranking of OA was more severe in Aarhus (*p* < 0.001, Kruskal–Wallis test) (Table 3). Radiographic classifications and urbanization level were not associated (*p* > 0.4, Kruskal–Wallis tests). Males had significantly more advanced OA than females using all three radiographic evaluation methods (*p* ≤ 0.01, Mann–Whitney U). On a hospital level, this difference was significant in Copenhagen (*p* ≤ 0.03), partly significant in Aarhus (*p* = 0.009–0.09), and not significant in Aalborg (*p* = 0.9).

### Incidence of surgery and implant selection

In Capital Region, the incidence of primary KA surgery in patients aged 60–79 years in 2017 was 28% higher than in Central Denmark Region and 15% higher than in North Denmark Region (Table 5). 22 surgeons treated the SPARK patients: 4 in Aarhus, 6 in Aalborg and 12 in Copenhagen. All surgeons were exclusively occupied with joint replacement surgery, except for five surgeons in training programs, who were responsible for fewer than six operations each and were evenly distributed among hospitals. With the exception of one surgeon in each hospital, the staffs had remained stable over the preceding years.

**Table 5:** Regional incidence of primary knee arthroplasty per region in the year 2017

	Central Denmark Region	North Denmark Region	Capital Region	p^1^
Regional revision rate	Low	Low	High	
SPARK example (hospital)	Aarhus	Aalborg	Copenhagen	
Incidence per 100.000 inhabitants				
All patients aged > 40 years	235	276	285	**< 0.001**
Subgroup: ages 60–79 years	416	463	534	**< 0.001**

Bold figures denote significant values of *p* < 0.05

^1^Chi-Square test

Implant selection varied widely across hospitals (Table 3). Overall, MUKA patients (67.0 ± 9 y) were 1.7 years younger (CI 0.6–3, *t* test) than TKA patients (68.8 ± 9 y), more likely to be male (52 vs. 44%, *p* = 0.01, Chi-Square), had a lower BMI (28.1 vs. 29.2 kg/m^2^, i.e., − 1.1 kg/m^2^ CI − 1.7−(− 0.5), t-test) and reported 1.4 points higher OKS (24.3 vs. 22.9, CI diff. 0.6–2, *t* test) and 3.9 (CI 1–6, *t* test) points better general health (EQ-VAS 64.5 vs. 60.6). In Aarhus, which had the highest frequency of MUKA use (40% MUKA, 51% TKA), there was no difference in age, sex or BMI between two patient groups (Table 6). In contrast, group differences were more pronounced in self-reported health (EQ-VAS), global knee anchor and patient-reported knee range of motion, e.g., preoperative flexion was 0.5 points better in MUKA patients (equivalent to approximately 5–10 degrees) [21].

**Table 6:** Characteristics of TKA vs. MUKA patients in hospitals grouped by frequency of MUKA use

Sample	Aarhus		*p*	Aalborg and Copenhagen		*P*
	TKA	MUKA		TKA	MUKA	
*n* (% of TKA + MUKA group)	164 (56)	129 (44)		895 (81)	207 (19)	
Demographics						
Age	67.0 ± 9	67.4 ± 9	0.7^*1*^	68.9 ± 9	66.7 ± 9	**0.001**^*1*^
Male sex (%)	76 (46)	59 (46)	1^*2*^	393 (44)	117 (57)	**0.001**^*2*^
BMI (kg/m^2^)	28.8 ± 5	28.2 ± 5	0.3^*1*^	29.2 ± 5	28.0 ± 4	**0.002**^*1*^
UCLA Activity Scale	4.6 [4] ± 2	5.0 [5] ± 2	**0.04**	4.6 [4] ± 2	5.1 [4] ± 2	**0.001**
Patient-reported outcomes						
OKS	22.8 ± 8	24.1 ± 6	0.1^*1*^	23.0 ± 7	24.4 ± 7	**0.005**^*1*^
Global knee anchor	24 ± 17	29 ± 17	**0.01**	28 ± 17	31 ± 18	0.06
EQ-VAS	59 ± 22	65 ± 20	**0.02**^*1*^	61 ± 22	64 ± 21	**0.046**^*1*^
EQ-5D-5L Index	0.58 ± 0.2	0.61 ± 0.1	0.06^*1*^	0.58 ± 0.2	0.61 ± 0.1	**0.008**^*1*^
Flexion (CKRS)	4.6 [5] ± 1	5.1 [5] ± 1	** < 0.001**^*1*^	4.8 [5] ± 1	5.1 [5] ± 1	**0.008**^*1*^
Deficit (CKRS 0–4) (%)	63 (38)	24 (19)	** < 0.001**^*2*^	268 (30)	47 (23)	**0.045**^*2*^
Extension (CKRS)	3.1 [3] ± 1	3.7 [4] ± 1	**0.009**^*1*^	3.4 [3] ± 1	3.7 [4] ± 1	**0.007**^*1*^
Deficit (CKRS 0–3) (%)	105 (64)	60 (47)	**0.004**^*2*^	529 (59)	84 (41)	** < 0.001**^*2*^
Radiographic assessments of knee OA						
K-L grade ≥ 2 (%)	99 (98)	103 (98)	1.000^*2*^	618 (94)	167 (90)	**0.09**^*2*^
K-L grade ≥ 3 (%)	81 (80)	97 (92)	**0.02**^*2*^	527 (80)	146 (79)	0.7^*2*^
Ahlbäck score ≥ 2 (%)	74 (74)	80 (76)	0.8^*2*^	444 (68)	106 (57)	**0.01**^*2*^
Ahlbäck score ≥ 3 (%)	41 (41)	20 (19)	**0.001**^*2*^	220 (33)	24 (13)	** < 0.001**^*2*^
Surgeons' ranking (mean [IQR]))	315 [118; 654]	486 [279; 463]	**0.003**^*3*^	518 [253; 809]	710 [505; 887]	** < 0.001**^*3*^

Bold figures denote significant values of *p* < 0.05

Test results refer to comparisons within the hospital group. When nothing else is stated, means [and medians] ± SD are reported. For some non-parametric variables, means and ± SD have been reported to aid comparison

*TKA* Total Knee Arthroplasty. *MUKA* Medial Unicompartmental Knee Arthroplasty. *BMI* Body Mass Index. UCLA Activity Scale range 1–10 (10 most active). *OKS* Oxford Knee Score (0–48 version, 48 best). *Global knee anchor* Patients’ overall knee assessment (VAS 0–100, 100 best). *OA* osteoarthritis. *K-L* Kellgren-Lawrence. *CKRS* Copenhagen Knee ROM Scale Flexion 0–6 (6 is max), Extension 0–5 (5 is max), see text for details. *Surgeons’ ranking* radiographic knee OA severity, total range 1- 1051 (1 is most severe)

Statistical tests: ^1^Unpaired *t* test. ^2^Chi-square test. ^3^Mann–Whitney *U* test

## Discussion

All hospitals had comparable preoperative PROM scores, indicating comparable symptom states prior to primary knee arthroplasty. Particularly four findings were unexpected in relation to commonly accepted revision risk factors: A very high percentage of patients from a low-revision hospital (Aarhus) were treated with unicompartmental implants, patients in both low-revision hospitals were younger than those in the high-revision hospital (Copenhagen), and the mean BMI and percentage of male patients was greater in one low-revision hospital (Aalborg) than elsewhere. Based on the literature, a higher risk of revision was expected in these four situations [9, 11, 29, 39]. In contrast, the more severe radiographic knee OA in a low-revision hospital (Aarhus) was consistent with previous findings [4, 6, 35]. The summarized findings show that the historical differences in revision rates among the three centres studied cannot easily be explained by variations in preoperative patient characteristics (Table 7).

**Table 7:** Summary of main findings

Predictor	High vs low-revision hospitals
PROMs (OKS, UCLA, etc.)	No difference
Incidence of knee arthroplasty	High incidence in high-revision hospital area
Radiographic OA	More severe OA in one of two low-revision hospitals
Age	Higher in the high-revision hospital
Sex	More males in one of two low-revision hospitals
BMI	Higher in one of two low-revision hospitals
Self-reported anxiety and depression^1^	Lower in one of two low-revision hospitals
Implant type	More unicompartmental implants in one low-revision hospital

*PROMs* Patient-reported outcome measures. *OKS* Oxford Knee Score *UCLA* UCLA Activity Scale. *OA* osteoarthritis. *BMI* Body Mass Index

^1^EQ-5D-5L item

### Strengths and limitations

Due to the observational nature of the study, causal conclusions cannot be drawn. Also, when a number of parameters are investigated, some significant differences will be discovered that are not necessarily reproducible or clinically important, as may be the case for e.g. the small difference in knee extension [21]. Similarly, the magnitude and clinical relevance of hospital differences in age or BMI may be debatable.

It is an important strength that the results were based on patients treated in routine clinical settings. Surgeons were not aware of any changes to patient selection practices during (or leading up to) the study period, so it was assumed that the study reflected standard hospital practice. However, the differences in treatment routines across hospitals introduce a massive amount of bias that cannot be compensated for through analyses, the most important probably being implant selection; Aarhus offered unicompartmental implants to 49% of all patients and only here, the choice between TKA and MUKA did not appear to be influenced by age, sex or BMI, an approach supported by recent literature [20, 25].

Response rates were relatively high. Numerous PROMs from nine out of ten participants in conjunction with radiographic OA classifications should provide a valuable reference set for future comparisons [42]. However, not all potential candidates were included, inevitably resulting in bias. To make the inclusion process feasible, no information was collected regarding patients who were not invited or declined participation and the reasons why. The surgeons and medical students in charge of patient recruitment reported that inclusion was occasionally overlooked or not prioritized, but patients were eager to participate. One could argue that the electronical collection of PROMs posed a threat to patient representation. However, Danish citizens are among the most IT-literate in Europe (2 out of 3 Danish citizens > 65 years used the internet daily in 2017) [45] and knee OA patients have previously preferred electronic questionnaires over paper ones [10]. Though the demography of the SPARK cohort largely resembled the surgical population of 2017 and the underlying hospital differences in demography were reflected in the SPARK cohort, males and young patients *were* overrepresented in the study. Participants without email address were 8 years older than others and were only allowed participation in the 6 of the 16 inclusion months. Therefore, it must be assumed that some of the oldest and possibly least resourceful patients were excluded, resulting in additional inclusion bias. Objective information regarding comorbidity or socioeconomic factors could have revealed important hospital differences in baseline health [40]. As a proxy of socioeconomic factors, 10% of men and 8% of women in age group 65–74 years reported daily smoking; this proportion was lower than the 17% and 14% reported in the National Health Profile 2018 [12]; however, smoking *is* associated to lower risk of OA (Relative Risk 0.80) [16].

In Aalborg, the low inclusion rate threatened the generalizability of results. A low level of self-reported anxiety and depression (especially among females) here may be a reflection of daily practice or may result from inclusion bias. The high proportion of males among patients undergoing KA surgery was a general tendency in Aalborg.

In this study, urban–rural variations in radiographic classifications were minimal. This may be due to the relatively small geographical distances in Denmark: almost all citizens live within a 1.5 h drive of a KA centre [27, 34]. In Aarhus, which is located in a region with a KA incidence 18–22% lower than the Capital Region, fewer patients with mild degrees of radiological OA underwent surgery. This would suggest that not all patients in Capital Region would have been offered (or accepted) primary KA surgery if they had lived in the Central (or North) Denmark Region. Utilization of primary KA is known to vary across economies and countries, for example by a factor of ten between countries in the Organization for Economic Co-operation and Development (OECD) alone [27, 29]. In welfare countries, the utilization of KA varies by a factor of two [26], and there are large regional variations within countries (Finland 1.6, Germany 1.8 and Spain factor of 27) [8, 19, 34]. In this light, the Danish variation in KA incidence by a factor of 1.3 is negligible. Regional variations in the threshold for primary KA surgery are not necessarily explained by the actions of knee surgeons alone [38]; expectations for surgery and risk aversion among patients, physicians and other caregivers (e.g. physiotherapists) the number of patients admitted for orthopaedic evaluation [18]. Therefore, the optimal comparison of patient selection should also include knee OA patients treated outside of hospitals and with non-surgical methods.

## Conclusions

The observed hospital variations in patient selection prior to primary knee arthroplasty were not associated with well-known revision risk factors to an extent that could reasonably explain the persistent differences in revision rates among three Danish high-volume hospitals. These baseline data provide the basis for comparing postoperative outcomes within the same cohort.

## Footnotes

1 Based on validation studies, “flexion deficit” (flexion scores 0–4) would identify 95% of patients with passive flexion below 100° (sensitivity) and exclude 81% of those with flexion above that limit (specificity). Similarly, “extension deficit” (extension scores 0–3) would identify 78% of patients with passive extension worse than 10° and exclude 70% of those with extension better that 10°.
